# Evaluating NGS-generated contig accuracy for detection and assignment of PRRSV against a reference genome in experimentally mixed PRRSV infection samples

**DOI:** 10.3389/fvets.2026.1875367

**Published:** 2026-09-22

**Authors:** Mariamawit Mohammed, Daniel Linhares, Hao Tong, Jianqiang Zhang, Philip Gauger, Michael Zeller, Ganwu Li, Giovani Trevisan

**Affiliations:** Veterinary Diagnostic and Production Animal Medicine, Iowa State University, Ames, IA, United States

**Keywords:** contigs, epidemiology, NGS, processing fluids, PRRSV management and control, PRRSV-2, sequencing

## Abstract

Mixed infections with multiple Porcine reproductive and respiratory syndrome virus (PRRSV) are common in swine production and complicate diagnostic interpretation, particularly when whole genomes cannot be recovered through next-generation sequencing (NGS). This study evaluated the ability of NGS to detect and characterize multiple PRRSV-2 strains present in controlled viral mixtures that varied in strain number, viral load, and proportional abundance, ultimately providing guidance for interpreting NGS outputs. Nine PRRSV-2 viruses, including three wild-type viruses and six commercial vaccine viruses, were spiked into processing fluid at Ct values of 21 and 27 to generate standardized combinations that reflect field conditions. Across all mixtures, NGS resulted in contigs that identified the correct strain composition in 89.7% of samples. NGS detected virus present at 10% concentration in 95.8% (23 of 24) of samples containing two viruses diluted at a 1:9 mixture. Contig nucleotide identity to reference strains decreased as viral load declined, yet accurate strain assignment was still achieved in most cases using a threshold of at least 98% nucleotide identity for high-concentration samples and at least 96% for lower-concentration samples. Detection failures occurred in 10.3% (8 of 78) of mixtures, 75% (6 of 8) of which contained four or more strains, indicating reduced NGS sensitivity under highly complex conditions with the same viral species. In different instances, NGS failed to detect MLV vaccines when 2 or more wild-type viruses were present. In 0.7% (8 of 1,147) of contigs, assembly artifacts were identified that proved to be artificial recombinant contigs, and 0.61% (7 of 1,147) were classified as inconclusive because they matched two highly similar vaccine strains at greater than 98% nucleotide identity. These results demonstrate that meaningful interpretation of PRRSV variant composition is possible from partial genome recovery, provided appropriate identity thresholds are applied and reference genetic sequences are available for comparison. The findings provide practical guidance for diagnostic laboratories and veterinarians who rely on NGS-derived contigs for PRRSV classification in field samples.

## 1 Introduction

In 1987, a previously unrecognized condition termed mystery swine disease was reported in the United States, characterized by abortion and respiratory distress (1, 2). Around the same time, similar clinical syndromes were observed in Europe, with reports of high mortality, abortion, stillbirths, and an increased number of mummified fetuses (3). In 1991 and 1992, the etiologic agents of PRRSV-1 and PRRSV-2 were identified following the successful isolation of Porcine Reproductive and Respiratory Syndrome Virus (PRRSV) from tissues collected from clinically affected piglets and sows (4, 5). The isolated virus was subsequently used to experimentally reproduce the disease in naïve pigs and was re-isolated from those animals (6). Since then, the disease was renamed porcine reproductive and respiratory syndrome (PRRS) (7) and is now found in most major swine-rearing countries worldwide. PRRSV is an enveloped, positive-sense, single-stranded RNA virus with a ~15 kb genome that contains at least 10 open reading frames (ORFs) (8). The virus belongs to the family *Arteriviridae* and is classified into two distinct species, *Betaarterivirus europensis*, commonly known as PRRSV-1 or European type, and *Betaarterivirus americense*, commonly known as PRRSV-2 or North American type (9).

PRRSV is one of the most economically damaging swine diseases in the United States and is estimated to cost the industry $1.2 billion each year (10). This substantial financial burden is not unique to the US, as comparable economic impacts have been documented globally. In the Netherlands, a 2012 study conducted with sow herds reported an average loss of €126 ($144) per sow within the first 18 weeks following a PRRSV outbreak (11). In Germany, an economic analysis of 21 pig herds estimated a median annual loss of €74,181 ($84,776) per farm attributable to PRRS (12). Similarly, in China, the economic impact of PRRSV at the herd level has been estimated at ¥1,424.37 ($209) per sow (13).

Genetic investigation of PRRSV has relied heavily on sequencing specific genes using Sanger sequencing. The open reading frame-5 (ORF5) gene, which represents approximately 4% of the PRRSV genome, has been and is still used for genetic classification and phylogenetic categorization of PRRSV, leading to the development of multiple lineage, sub-lineage, and variant naming systems through the years (14, 15). Similarly, the ORF-7 gene, 2% of the 15k genome, has been used for diagnostic assays and molecular detection (16). However, as next-generation sequencing (NGS) and improved bioinformatics tools become more accessible (17), the swine industry is increasingly adopting whole-PRRSV genome sequencing (WGS) for PRRSV epidemiological investigations. Recovery of whole PRRSV genomes enables a better understanding of the virus and its evolution (18).

This diagnostic advancement using NGS creates a tremendous opportunity for the development of novel diagnostic techniques to assess the presence, absence, and understand the genetic characteristics of PRRSV and support disease management and control decisions. Despite the advancements in the NGS technology, wet lab procedures, and assembly procedure, only partial PRRSV genome fragments, as a series of gapped contiguous sequences, referred to as contigs, are routinely recovered when multiple PRRSV variants are in the sample or the viral load is low, i.e., high cycle threshold (Ct) values recovered through Reverse Transcription-Quantitative Polymerase Chain Reaction (RT-qPCR) testing (9). PRRSV exhibits an ever-expanding genetic diversity contributed by a fast evolutionary rate (19–21), the occurrence of frequent recombination events (22, 23), and the frequent herd co-infection with multiple PRRSV variants (9). Thus, in addition to the development of better NGS wet-lab procedures and assembling pipelines, there is an opportunity to fully leverage NGS-reported results for epidemiological investigations, even if only genome fragments are recovered, rather than resorting to additional retesting or resampling, equipping veterinarians and producers with knowledge of the PRRSV variant affecting their pig populations. Thus, the primary objective of this project was to establish guidelines for interpreting NGS-reported outcomes from samples containing mixed infections of multiple PRRSV-2 strains that yield contigs during the NGS assembly from raw reads. Specifically, this work aimed to establish evidence-based recommendations that can assist epidemiological investigations in interpreting NGS-assembled contig results, assessing the reliability of PRRSV strain identification, and to develop a guideline for result interpretation.

## 2 Materials and methods

### 2.1 Reference PRRSV isolates

Three PRRSV-2 wild-type viruses a Lineage 1A [L1A (65239GA)], a L1C.5 (01775GA), and a L1H (81793–6)) and five US commercially available PRRSV-2 MLV vaccines, namely Prevacent^TM^ PRRS (Elanco, Greenfield, IN, USA) (GenBank KU131568.1), Prime Pac^®^ PRRS RR (Merck Animal Health, Rahway, NJ, USA) (GenBank DQ779791.1), Fostera^®^ PRRS vaccine (Zoetis, Parsippany, NJ, USA) (GenBank AF494042.1), Ingelvac PRRS^®^ MLV (Boehringer Ingelheim, Duluth, GA, USA) (GenBank AF066183.4), and PRRSGard^®^ (Pharmgate, Wilmington, NC, USA), and the formerly commercialized, no longer in the US market, Ingelvac PRRS^®^ ATP (Boehringer Ingelheim, Duluth, GA, USA) (GenBank DQ988080.1) were used in this study.

The three wild-type PRRSV strains were selected because they represent the PRRSV sublineage groups most commonly detected in the United States in recent years (24). Each virus was isolated from field samples and further propagated in cell culture. The L1A isolate was grown in MARC-145 cells, and passage eight was used for this study. Similarly, the L1C.5 isolate was propagated in MARC-145 cells, with passage six used. The third isolate, L1H, was cultured in ZMAC cells, and passage four was used in the experiment. Four of the six vaccines (Prevacent, Ingelvac MLV, Fostera PRRS, and Prime Pac) were purchased directly from the market. Ingelvac ATP and PRRSGard were not available in the market at the time of the study, whereas Ingelvac ATP production and commercialization have been discontinued, and PRRSGard was temporarily unavailable. For Ingelvac ATP and PRRSGard, previously purchased, ultra-low-temperature (−80 °C) stored aliquots were used. The complete genome sequences of each of the nine viruses were sequenced using Illumina technology. The complete genomes of each reference strain were further used to compare nucleotides across all contigs assembled through NGS recovered from the performed dilutions (see section 2.1.4). The nucleotide identity at the whole-PRRSV genome level between the reference strains is presented in Table 1.

**Table 1:** Pairwise nucleotide identity matrix based on whole PRRSV genome for wild-type and modified vaccine PRRSV used as a reference genome.

Strain	L1A	L1C.5	L1H	Prevacent	Prime Pac	PRRSGard	Ingelvac MLV	Ingelvac ATP
L1C.5	88.96							
L1H	83.70	82.52						
Prevacent	83.73	82.79	85.08					
Prime Pac	82.63	82.25	81.22	82.71				
PRRS Gard	82.76	82.33	81.49	82.68	98.19			
Ingelvac MLV	83.08	83.11	81.62	83.68	93.09	92.35		
Ingelvac ATP	82.74	82.52	80.87	82.67	89.48	88.93	90.87	
Fostera	82.99	82.72	81.70	82.75	90.05	89.33	91.33	94.12

### 2.2 PRRSV reference strain characterization

Each virus isolate and vaccine sample was initially characterized by RT-qPCR and NGS to determine the Ct value and the whole PRRSV genome sequence. Samples were tested on a fee-for-service basis at the Iowa State University Veterinary Diagnostic Laboratory (ISU-VDL) for PRRSV-1 and PRRSV-2 RNA using commercially available and previously validated RT-qPCR assays (Applied Biosystems TaqMan kit for North American and European PRRSV detection, Thermo Fisher Scientific, Waltham, MA, USA) (25). All samples were considered positive for PRRSV RNA when the RT-qPCR Ct was below 37 (23). All samples were then submitted for NGS testing at ISU-VDL. Every lab-bench-produced virus mixture, described in the virus dilution section, was also submitted for RT-qPCR to confirm the intended concentration was achieved and for NGS testing. Laboratory personnel were blinded to the study design and testing procedures. NGS assembly procedures and pipelines were not part of the evaluation and analysis of this study; instead, the study focused on evaluating assembled contigs.

### 2.3 Diluent selection and sample sources

Due to the increased usage of processing fluid (PF) as a sample type for PRRSV monitoring and surveillance in breeding herds (26, 27), this sample type was selected as the diluent for this study. Three PRRSV-naïve multiplication farms were identified, and processing fluid samples were collected weekly over a 2-week (2-batch) period to obtain a sufficient volume of 850 ml. All samples were tested by RT-qPCR and ELISA to confirm the absence of PRRSV or evidence of an immune response indicative of prior PRRSV exposure. The testing was done separately by farm (3 farms) and by week (2 weeks). After confirming all processing fluid samples were negative by both RT-qPCR and ELISA, the samples were centrifuged at 1,400 × g for 5 min. The resulting supernatant was transferred into a 1,000 ml tube to ensure thorough homogenization. The homogenized fluid was then aliquoted into 50 ml tubes and stored at −20 °C until further use as a diluent (see section 2.1.3).

### 2.4 Virus dilution and mixing procedure

All nine PRRSV-2 viruses, including three wild-type isolates and six vaccines, were initially characterized by RT-qPCR and NGS to determine Ct values and complete genome sequences. Following characterization, each virus was diluted using RT-qPCR and ELISA PRRSV-negative processing fluid to adjust and achieve target Ct values of 21 (Table 2) and 27 (Table 3). The volume of diluted virus preparations varied according to the desired final Ct value combination, ranging from 3,000 μl to 40,000 μl for each Ct target. Tables 2, 3 provide detailed information on the original Ct values, the volume prepared, the amount of processing fluid added, and the final Ct values achieved for the Ct 21 and Ct 27 target concentrations, respectively.

**Table 2:** PRRSV-2 virus dilutions spiked in PRRSV-negative processing fluids (PF) samples targeting a Ct value of 21.

Strain	Starting Ct-value	Volume of virus (μl)	PF volume (μl)	Final volume (μl)	Final Ct-value
L1A	12.6	236.8	39,762	40,000	21.5
L1C.5	14.9	1,166.3	38,832	40,000	21.2
L1H	17.3	6,155.7	33,844	40,000	21.1
Ingelvac ATP	19.6	2,273.6	726	3,000	20.9
PRRSGard	20.8	3,000	0	3,000	20.8
Prevacent	14	79	4,921	5,000	22.9
Ingelvac MLV	16.6	473.7	4,526	5,000	22.2
Fostera PRRS	18	1,250	3,750	5,000	22.2
Prime Pac	19.3	1,846.7	1,153	3,000	20.8

**Table 3:** PRRSV-2 virus dilutions spiked in PRRSV-negative processing fluids (PF) samples targeting a Ct value of 27.

Strain	Starting Ct-value	Volume of virus (μl)	PF volume (μl)	Final volume (μl)	Final Ct-value
L1A	21.5	625	39,375	40,000	27.4
L1C.5	21.2	625	39,375	40,000	27.3
L1H	21.1	625	39,375	40,000	27.3
Ingelvac ATP	20.9	46.8	2,953.1	3,000	28.6
PRRSGard	20.8	46.8	2,953.1	3,000	28.3
Prevacent	22.9	78.1	4,921.9	5,000	27.8
Ingelvac MLV	22.2	78.1	4,921.9	5,000	28.7
Fostera PRRS	22.2	78.1	4,921.9	5,000	29.2
Prime Pac	20.8	46.8	2,953.1	3,000	27.9

### 2.5 Mixtures of PRRSV isolates

All nine PRRSV-2 lineages, including three wild-type viruses (L1A, L1C.5, and L1H) and six commercial vaccines, were used to create a one-time virus mixture that simulated a co-infection combination, potentially reflecting field co-infections observed in the United States. The mixtures not only mimic the type of virus that could be present but also the abundance of different viruses in the sample by adjusting to two viral loads measured at Ct 21 and Ct 27. A one-time virus mixture was prepared due to budget constraints and to simulate a real-world scenario in which veterinarians submit samples for testing without duplicates.

Pairwise mixtures of the three wild-type strains were prepared to generate two virus mixtures. Each pair, e.g., L1A + L1H, was mixed under two conditions. In the equal Ct condition, e.g., Ct 21, both isolates were adjusted to the same Ct value and combined at five volumetric ratios of 1:9, 3:7, 1:1, 7:3, and 9:1. Fifteen mixtures were prepared at Ct 21 and 15 at Ct 27. In the unequal Ct condition, one isolate was adjusted to Ct 21 and the other to Ct 27, and the same five ratios, i.e., 1:9, 3:7, 1:1, 7:3, and 9:1, were used, resulting in an additional 30 mixtures (Figure 1A). A total of 60 two-virus wild-type mixtures were produced.

**Figure 1:**
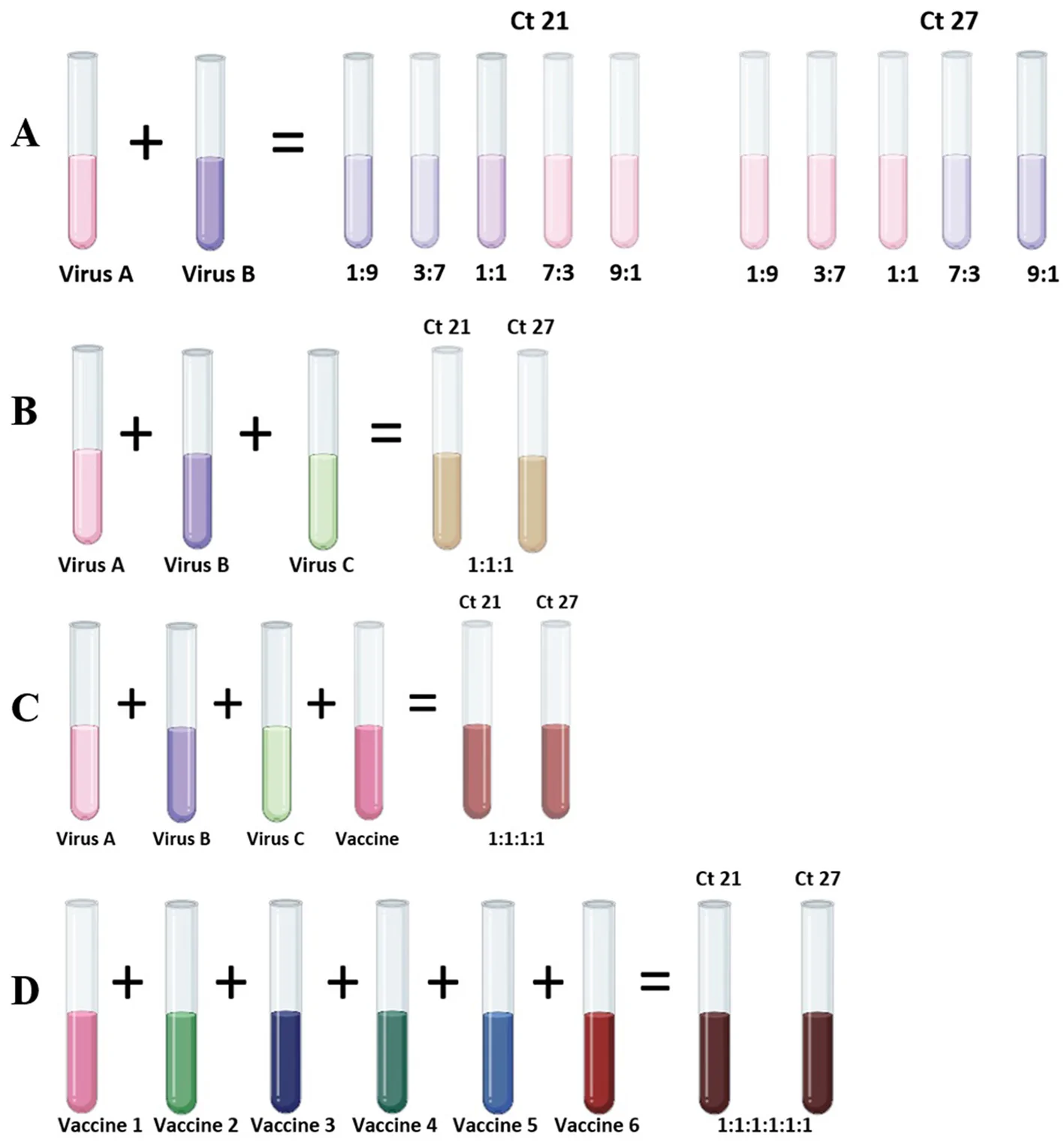
**(A)**. Schematic representation of two wild-type PRRSV mixing designs. Pairs of PRRSV strains (L1A + L1C.5, L1A + L1H, and L1C.5 + L1H) were combined at two identical Ct levels and at two different Ct levels for both viruses in the mix (21 and 27) using five volumetric ratios (1:9, 3:7, 1:1, 7:3, and 9:1). **(B)** Schematic representation of the three-virus mixing design. The three virus mixes were combined at two Ct levels in a 1:1:1 ratio. **(C)** Schematic representation of the four-virus mixing design. The four viruses were mixed in a 1:1:1:1 ratio at two Ct levels. **(D)** Schematic representation of the six-virus mixing design. The six viruses were mixed in a 1:1:1:1:1:1 ratio at two Ct levels.

Three virus mixtures containing all three wild-type strains were combined in a 1:1:1 ratio. Two sets were prepared, one using aliquots adjusted to Ct 21 and one using aliquots adjusted to Ct 27 (Figure 1B). A parallel set of three virus mixtures was prepared by combining the vaccines Prevacent, Ingelvac MLV and Fostera PRRS, which are the most commonly recovered by PRRSV ORF5 Sanger sequencing in the United States (24), in a 1:1:1 ratio at Ct 21 and at Ct 27.

Four virus mixtures were generated by combining each of the three wild-type strains with a single vaccine. For each of the six vaccines, a mixture contained L1A, L1C.5, L1H, and the corresponding vaccine at equal volumetric proportions (1:1:1:1). This procedure was carried out at Ct 21 and at Ct 27 (Figure 1C).

Two six-virus mixtures were created by combining all six commercial vaccines in equal proportions. Each mixture consisted of the six vaccines in a 1:1:1:1:1:1 ratio, prepared separately at Ct 21 and Ct 27 (Figure 1D).

### 2.6 Next-generation sequencing procedures

Total nucleic acid extraction and sequence library preparation were conducted as previously described (9, 18). In brief, nucleic acids were extracted using the MagMAX™ Pathogen RNA/DNA Kit (Thermo Fisher Scientific, USA). Double-stranded cDNA was synthesized using the NEXTflex™ Rapid RNA-Seq Kit (Revvity Health Sciences, Inc., USA). Sequencing libraries were constructed using the Illumina DNA Prep kit (Illumina, USA) with dual indexing. Fluorometer Qubit 2.0 (Life Technologies, Carlsbad, CA) was used to determine the concentration of the sequencing library, which was then normalized to a concentration of 2 nM. The pooled libraries were sequenced on an Illumina MiSeq i100 platform at the NGS section in the ISU-VDL on a fee-for-service basis, with a 600-Cycle Reagent Kit (Illumina) to generate 300-base-pair paired-end reads by following standard Illumina protocols (28). The team performing the NGS test and the sequence assembly analysis were blinded to the study methods, the PRRSV strains present in the samples, and the mixed-infection nature of the samples.

Raw Illumina paired-end reads were assessed for quality using FastQC v0.11.9, and quality reports were summarized with MultiQC v1.11. Adapter removal and quality trimming were performed using Trimmomatic v0.39 (29). Adapter sequences were removed using the ILLUMINACLIP module (seed mismatches = 2, palindrome clip threshold = 30, simple clip threshold = 10, minimum adapter overlap = 8, keep both reads = true). Low-quality bases (Phred score < 3) were trimmed from both the 5′ and 3′ ends (LEADING:3 and TRAILING:3), followed by sliding-window trimming (SLIDINGWINDOW:4:20), which removed sequence once the average quality within a 4 bp window fell below Q20. Reads shorter than 36 bp after trimming (MINLEN:36) were discarded before downstream analysis.

Trimmed reads were first aligned to the host reference genome [GenBank GCF_000003025.6 (Sscrofa11.1)] using HISAT2 v2.2.1 with the sensitive flag, and host-derived reads were removed prior to *de novo* assembly using SPAdes v3.13.2 (18, 30) and ABySS v 2.2.4 following procedures previously described (18). No minimum contig length threshold was applied during assembly. Contigs generated from both assemblers were compared against the NCBI nucleotide database using BLASTn v2.15.0 to identify the closest reference genomes and to distinguish target viral contigs from non-target sequences and chimeric assemblies. Candidate viral contigs were subsequently aligned to the corresponding reference genome, and sequencing reads were mapped back to the assembled contigs using BWA v0.7.17. Alignment files were processed with SAMtools v1.6 and visually inspected in Integrative Genomics Viewer v2.10.2 to evaluate read-mapping patterns, coverage consistency, and potential assembly errors. Contigs were classified based on sequence similarity, read-mapping evidence, and manual inspection. Validated viral contigs were defined as those with a BLAST best match to PRRSV and continuous, well-supported read alignments across the contig. Contaminant contigs were identified based on BLASTn matches to non-target organisms or host sequences. Chimeric contigs were identified by abrupt changes in BLAST sequence similarity, split or discordant alignments, inconsistent read-mapping patterns, or abrupt shifts in coverage observed during IGV inspection. Assembly artifacts were defined as contigs showing poor or inconsistent read support, isolated low-coverage regions, or other evidence of misassembly during manual inspection. Missing genomic regions were defined as those that could not be confidently assembled due to insufficient supporting reads. Following manual curation, validated viral contigs were used as references for reference-guided assembly to generate draft viral genomes. Because the study used processing fluid spiked with PRRSV rather than cultured viral isolates, viral abundance varied substantially among samples. Consequently, no fixed minimum read depth or genome coverage threshold was applied during consensus generation. Instead, assembled genomes were evaluated for concordance among *de novo* assemblies, reference-guided assemblies, BLAST results, read-mapping support, and manual inspection in IGV to ensure assembly accuracy.

Whole genomes or genome fragments reported by the NGS department were used for downstream analysis to simulate the same scenarios as a field veterinarian would encounter when submitting a sample and receiving a result and report. This study used NGS-assembled genomes for the epidemiological investigation of the detected virus. A contig mentioned outside section 2.1.5 refers to an NGS-assembled genome fragment, and a whole genome refers to the PRRSV NGS-assembled genome, which contains all non-structural ORFs (ORF1a and ORF1b) and the ORF2-ORF7 structural PRRSV genome regions. NGS-assembled contigs were used for this analysis. For each sample, NGS-reported contigs were pairwise aligned with the reference whole-PRRSV genome of the virus used to produce the dilution and expected to be recovered, and the percent nucleotide identity for each virus was calculated as the proportion of identical sites in the aligned region.

## 3 Results

Nine well-characterized PRRSV-2 viruses, comprising three wild-type isolates propagated in cell culture and six commercially available MLV vaccine strains obtained directly from vaccine vials, were spiked in PRRSV-negative processing fluid samples and used to simulate mixed infections involving wild-type plus wild-type, wild-type plus vaccine virus, and vaccine plus vaccine virus combinations. The nine viruses were mixed in different proportions with 2, 3, 4, and 6 virus combinations and two Ct values each. In total, 78 PRRSV-2 mixtures were sequenced using NGS (Table 4), with no virus having 100% rate of detection (Table 5), 1,147 contigs were reported (Table 6), ranging from 41 to 8,989 nucleotides in length (Supplementary Table 1). No complete PRRSV whole genomes were recovered from any of the mixtures.

**Table 4:** Summary of the NGS results for the 78 viral mixes based on Ct value and number of virus present in the dilution.

Mixture type	Number of samples	Rate of detection
Two–virus mix		
21 Ct		
Ref. virus ≥ 98% nt identity	15	100
27 Ct		
Ref. virus ≥ 98% nt identity	12	80.0
95–98% nt identity	1	6.6
Artificial recombinant contig	1	6.6
Contamination present	1	6.6
Ct 21 and 27 mixed		
Ref. virus ≥ 98% nt identity	19	63.3
95–98% nt identity	3	10.0
Artificial recombinant contig	2	6.6
Contamination present	3	10.0
Missing virus	2	6.6
95–98% nt identity and artificial recombinant contig	1	3.3
Three–virus mix		
21 Ct		
Ref. virus ≥ 98% nt identity	2	100
27 Ct		
Ref. virus ≥ 98% nt identity	2	100
Four–virus mix		
21 Ct		
Ref. virus ≥ 98% nt identity	5	83.3
Missing virus	1	16.7
27 Ct		
Ref. virus ≥ 98% nt identity	2	33.3
95–98% nt identity	1	16.7
Missing virus	3	50.0
Six–virus mix		
21 Ct		
Ref. virus ≥ 98% nt identity	0	0.0
Missing virus and inconclusive contigs ^a^	1	100
27 Ct		
Ref. virus ≥ 98% nt identity	0	0.0
Missing virus and inconclusive contigs ^a^	1	100

ref. = reference, nt = nucleotide. ^a^In two samples containing both Prime Pac and PRRSGard, seven assembled contigs could not be conclusively assigned because they exhibited high sequence similarity to both vaccine strains due to the extensive genomic similarity shared between them.

**Table 5:** Summary of the rate of detection for the nine viruses included in the study.

Strain	Total number of samples	Correctly detected	Rate of detection
L1A	54	52	96%
L1C.5	54	53	98%
L1H	54	52	96%
Ingelvac PRRS ATP	4	3	75%
PRRSGard^a^	4	3	75%
Prevacent	6	4	66%
Ingelvac MLV	6	4	66%
Fostera PRRS	6	3	50%
Prime Pac^a^	4	2	50%

^a^In two samples containing both Prime Pac and PRRSGard, seven assembled contigs could not be conclusively assigned because they exhibited high sequence similarity to both vaccine strains due to the extensive genomic similarity shared between them.

**Table 6:** Summary table of description of the contigs recovered and its classification according to nucleotide (nt) identity.

Ct value of the individual virus in the mix	# recovred	# ≥98% nt identity	# 96–98% nt identity	# with artificial recombinant contig	# contaminants	# inconclusive
21 Ct (24 mixes)	456	451	0	0	0	5
27 Ct (24 mixes)	219	213	2	1	1	2
21 Ct and 27 Ct mixed (30 mixes)	472	448	6	7	11	0

Overall, 73.1% (57 of 78) of the mixtures yielded all contigs that matched the reference genomes in the samples with at least 98% nucleotide identity, 7.7% (6 of 78) matched the reference genomes in the samples with 95 to 98% nucleotide identity, the remaining 19.2% (15 of 78) of the mixtures contained contigs that mismatched the reference strains, had missing virus, or inconclusive contigs (Table 4). These issues were further investigated, and they included contigs with low sequence identity (< 95%) to any reference virus in the mix, assembly artifact contigs, contamination, and cases in which a reference virus known to be present in the mixture but was not recovered by sequencing and was therefore classified as a missing virus. In 13.3% (2 of 15) with inconclusive results, contigs matched both the Prime Pac and the PRRSGard vaccine on the non-structural portion of the genome. Since Prime Pac and PRRSGard share >99.5% nucleotide identity in the non-structural portion, this creates an impediment to distinguishing them using assembled contigs

Six of the eight mixtures with missing virus were mixtures of four or more viruses (Table 4). In three samples containing four virus mixes, NGS recovered contigs for only three of the viruses and failed to detect the fourth (Tables 7, 8). A similar pattern occurred in another mixture where contigs were generated for only two viruses, and the remaining two were not recovered (Table 8). In the six-vaccine virus mixtures, NGS produced contigs for only Ingelvac ATP, PRRSGard and five contigs that are inconclusive between Prime Pac and PRRSGard strains at Ct 21. At Ct 27, NGS recovered contigs for Prevacent and two contigs inconclusive between Prime Pac and PRRSGard (Table 4). One sample containing four viruses at Ct 21 initially appeared to be missing one virus; however, following retesting, contigs corresponding to all four viruses were recovered.

**Table 7:** Genome coverage of three wild–type PRRSV lineages combined with one vaccine strain at Ct 21.

Vaccine in mixture	L1A coverage (%)	L1C.5 coverage (%)	L1H coverage (%)	Vaccine coverage (%)
Prevacent	54.0	69.8	71.2	Not detected
Ingelvac MLV	66.8	60.5	86.6	50.1
Fostera PRRS	62.3	79.2	75.3	27.1
Prime Pac	94.2	76.2	66.2	84.1
PRRSGard	60.5	72.7	68.3	93.0
Ingelvac ATP	63.1	74.7	87.0	68.6

**Table 8:** Genome coverage of three wild–type PRRSV lineages combined with one vaccine strain at a Ct 27.

Vaccine in mixture	L1A coverage (%)	L1C.5 coverage (%)	L1H coverage (%)	Vaccine coverage (%)
Prevacent	Not detected	13.9	27.1	26.5
Ingelvac MLV	10.7	27.8	21.4	11.4
Fostera PRRS	58.2	77.3	90.8	Not detected
Prime Pac	Not detected	Not detected	12.8	4.7
PRRSGard	12.2	25.4	27.4	32.4
Ingelvac ATP	10.8	13.3	14	11.1

In two virus mixtures prepared at the same viral load of Ct 21 and tested across five different ratios, all contigs (221 of 221) matched their respective reference genomes with more than 98% identity. Under the same conditions at Ct 27, 97.4% of contigs (114 of 117) were assigned correctly with 98% and above similarity, one contig 0.85% (1 of 117) matched at 96.6% identity, and 1.71% (2 of 117) were classified either as an assembly artifact or as a contaminant. For two virus mixtures prepared with different Ct values, 94.9% of contigs (448 of 472) matched their corresponding reference genome with at least 98% identity. Only 1.2% of contigs (6 of 472) showed identity between 96 and 98%, and four originated from the lower-concentration virus of Ct 27, and only 2 originated from the Ct 21 virus. The remaining 3.8% (18 of 472) were classified as assembly artifacts, that turned out to be artificial recombinant contigs, or contaminants (Table 6). In mixtures containing more than two viruses, 99.7% of contigs (336 of 337) aligned to their correct reference genome with at least 98% similarity. Of these, 2% (7 of 336) were inconclusive. Only a single contig (1 of 337), originating from a virus present at Ct 27, fell below this threshold and matched its reference at 97.92%.

Four mixtures that initially showed contamination with a viral lineage not included in the intended mix were retested as a post-testing confirmatory analysis. The retesting results were used solely to compare and contrast the original findings and were not included in the calculation of the reported performance metrics. Following re-analysis, one mixture showed no evidence of contamination, one mixture contained a single contaminant contig, and two mixtures exhibited an assembly artifact without any contaminant contigs.

### 3.1 Results across Two-, Three-, Four-, and Six-virus mixtures

In the two-virus mixtures prepared using the wild-type virus of the same Ct value, a total of 221 contigs were generated at Ct 21, with lengths ranging from 156 to 6,198 nucleotides. All contigs matched their expected reference genomes at ≥98% nucleotide identity, confirming that both wild-type components were accurately recovered in the assembly. An example of the result is presented in Figure 2.

**Figure 2:**
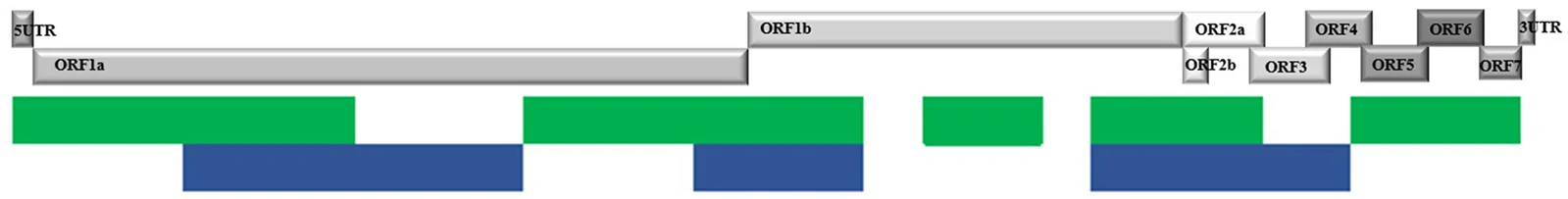
Two virus combinations of L1A and L1C.5, both standardized at Ct 21 and mixed at a 9:1 ratio. Five contigs (green) aligned to the L1A reference genome, and three contigs (blue) aligned to the L1C.5 reference genome. The regions of the PRRSV genome are indicated in the top gray box.

In the mixture containing wild-type PRRSV at Ct 21, genome coverage shifted consistently according to the mixing ratios. For the L1A plus L1C.5 combinations, coverage was the highest for each virus at 1:1 ratio (Figure 3). A similar pattern was observed in the L1A plus L1H mixtures. For both viruses, genome coverage changed gradually with varying proportions; however, when the proportion of L1H decreased from 30% to 10%, a pronounced decline in coverage was observed. For the L1C.5 plus L1H combinations, coverage again followed the expected direction of the input ratios. Coverage for L1H was higher at 1:1 or greater ratio, when L1H proportion was gradually reduced from 1:1 the genome coverage decreased gradually (Figure 3).

**Figure 3:**
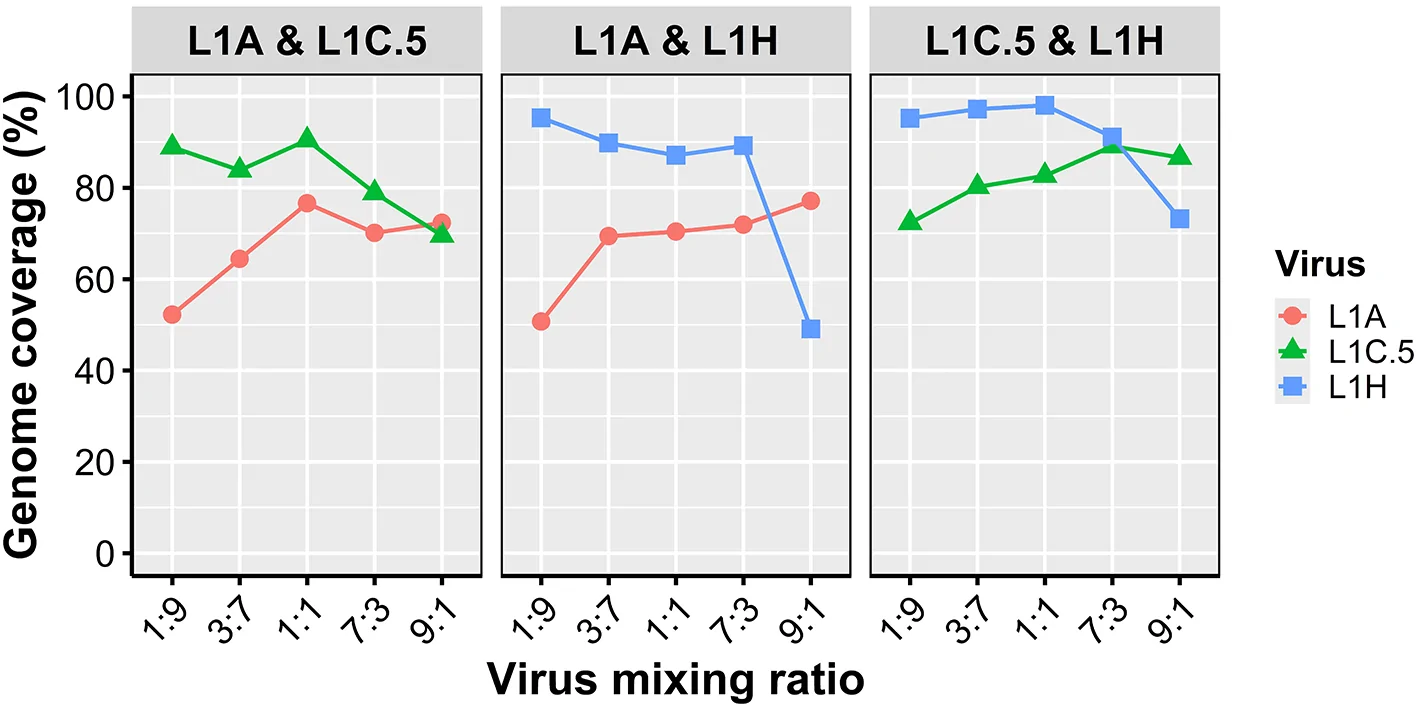
Genome recovery for different PRRSV strains from two virus mixes at Ct 21 across different mixing ratios. Each panel shows genome coverage for the two lineages included in the mixture, with points representing observed coverage and lines connecting ratios within each lineage. Virus mixture ratios are reported as first-listed virus:second-listed virus (e.g., 1:9).

In the mixture containing three wild-type PRRSV at Ct 21 each, a total of 19 contigs ranging from 231 to 5,266 nt were generated. All contigs matched their corresponding reference genomes with greater than 98% nucleotide identity. Genome coverage for L1A, L1C.5, and L1H was 67.3%, 79%, and 91%, respectively. In the mixture containing the three vaccine strains (Prevacent, Ingelvac MLV, and Fostera) at Ct 21 each, a total of 20 contigs ranging from 228 to 2,725 nt were generated. All contigs exhibited ≥98% nucleotide identity to their respective reference genomes. Genome coverage was 61.6% for Prevacent, 56.1% for Ingelvac MLV, and 48.1% for Fostera.

A total of 182 contigs were recovered from mixtures containing the three wild-type PRRSV (L1A, L1C.5, and L1H) plus one vaccine at Ct 21, with contig lengths ranging from 202 to 8,989 nucleotides. In the mixture containing the three wild-type strains and the Prevacent vaccine, no Prevacent contigs were recovered during initial testing (Table 7). However, upon re-testing of the sample, five contigs corresponding to Prevacent were identified. For comparison, when the retested result was included in the evaluation, a total of 23 contigs were recovered, representing all four reference genomes, with genome coverage of 42.2% for L1A, 72.0% for L1C.5, 62.3% for L1H, and 22.5% for Prevacent. Genome coverage results for the initial sample and the remaining four-virus mixtures at Ct 21 are summarized in Table 7.

In the six-vaccine mixture prepared at Ct 21, Prevacent, Ingelvac MLV, and Fostera PRRS were not detected, despite being included at equal proportions and identical Ct values. PRRSGard and Ingelvac ATP were successfully recovered and assembled with genome coverages of 20.9% and 54.9%, respectively. Prime Pac and PRRSGard share a nucleotide identity of 98.19%; accordingly, they have extensive overlapping genomic regions, e.g., ORF1a and ORF1b share 99.7%, nucleotide identity, and ORF2 99.6%, ORF3 94.5%, ORF4 to ORF7 89.2%, as a result, five assembled contigs placed across the non-structural region (ORF1a and ORF1b) of the PRRSV were classified as inconclusive because they matched both Prime Pac and PRRSGard with greater than 98% sequence similarity. However, two contigs derived from the structural region of the genome matched PRRSGard with 99.96% and 100% sequence similarity, compared with only 92.38% and 93.48% similarity to Prime Pac, enabling the assignment of PRRSGard in the sample based on the recovered contigs from the structural portion. Recovery of Prime Pac in this sample was inconclusive.

In the two virus mixtures prepared with the wild-type virus, both with a Ct value of 27, a total of 117 contigs were assembled, ranging in length from 134 to 2,285 nucleotides. Nearly all contigs matched their corresponding PRRSV reference genomes at or above the 98% nucleotide identity threshold. One contig of 265 nucleotides aligned at 96.6% identity, representing the only deviation from the expected match quality. In addition, one contig in the L1C.5 plus L1H mixture was an assembly artifact, leading to the misperception of an artificial recombinant contig virus (Figure 4). After being split cleanly into two segments, each contig aligned to the correct parental reference genome with more than 98% identity (Figure 4). Artificial recombinant contig events can be attributed to this scenario, since no viral propagation is expected to have occurred between dilution in processing fluids and NGS testing. This encountered issue underscores the need to confirm the presence of recombinant virus through other methods, such as virus isolation, plaque purification, targeted RT-PCR designed to target the breakpoints, and resequencing, to rule out artifacts in NGS-reported results. For the L1A plus L1C.5 mixture at a 7:3 ratio, one 204 nt contig was initially identified as a contaminant. The sample was retested, and no contaminant contig was recovered. As observed for the Ct 21 mixtures, genome coverage at Ct 27 generally shifted in accordance with the input ratios and reflected the underlying abundance of each virus in the template. However, for all dilutions at a Ct 27, the genome recovery was much lower than at a Ct 21.

**Figure 4:**
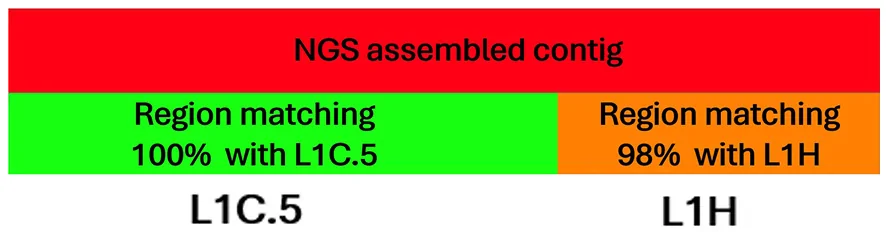
A 2,981 nucleotide base pair contig exhibiting artificial recombinant contig showed low nucleotide identity to either reference genomes (red). After splitting at the artificial recombination breakpoint, the contig yielded two fragments (1,915 nt and 1,066 nt) that matched L1C.5 and L1H with 100% and 98% nucleotide identity, respectively.

In the mixture containing wild-type PRRSV at Ct 27, Genome coverage generally followed the expected direction of the input mixing ratios across all pairwise combinations, although the magnitude and consistency of the response varied across mixes (Figure 5). In the L1A and L1C.5 mixtures, L1C.5 showed the highest coverage at the most L1C.5-dominant ratio and declined progressively as its proportion decreased, while L1A coverage increased modestly as its relative proportion increased peaking at the 70% proportion. In the L1A and L1H mixtures, L1H exhibited higher coverage when present at higher proportions, followed by a steady reduction as its contribution declined, whereas L1A coverage fluctuated slightly with a sharp reduction when raised from 10% to 30% after which it went back up with a steady reduction despite the proportion being raised. The L1C.5 and L1H mixtures displayed more variable behavior. L1C.5 coverage increased markedly as its proportion increased, reaching a peak when L1C.5 was 70% of the mix, while L1H coverage dropped sharply when reduced from 90% to 70% proportion. Overall, these patterns indicate that genome coverage broadly reflects input ratios, but at a high Ct value, coverage shows inconsistent trends (Figure 5).

**Figure 5:**
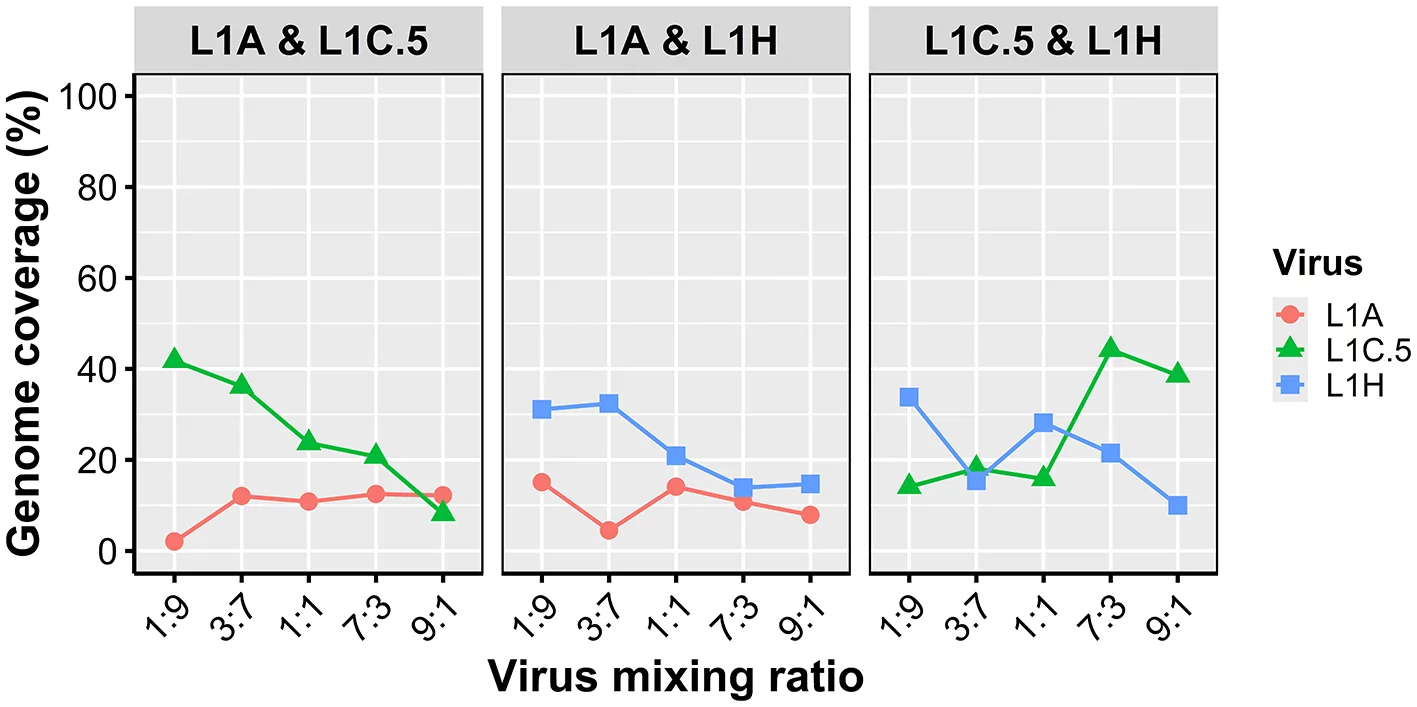
Genome recovery for different PRRSV strains recovered from two virus mixes at Ct 27 across different mixing ratios. Each panel shows genome coverage for the two lineages included in the mixture, with points representing observed coverage and lines connecting ratios within each lineage. Virus mixture ratios are reported as first-listed virus:second-listed virus (e.g., 1:9).

In the mixture containing the three wild-type PRRSV lineages (L1A, L1C.5, and L1H) prepared at Ct 27, a total of 18 contigs were generated, with lengths ranging from 200 to 1,771 nucleotides. All contigs matched their corresponding reference genomes at 98% or greater nucleotide identity. Genome coverage for L1A, L1C.5, and L1H was 29.9%, 33.1%, and 20%, respectively. In the mixture containing the three vaccine strains (Prevacent, Ingelvac MLV, and Fostera PRRS) prepared at Ct 27, a total of eight contigs were assembled, ranging in length from 225 to 1,781 nucleotides. All contigs exhibited 98% or greater nucleotide identity to their respective reference genomes. Genome coverage for the vaccine strains was 31.4% for Prevacent, 1.5% for Ingelvac MLV, and 11.5% for Fostera PRRS.

At Ct 27, a total of 73 contigs were recovered from mixtures containing three wild-type PRRSV lineages plus one vaccine, with contig lengths ranging from 201 to 4,145 nucleotides (Table 8). In the mixture containing the three wild-type strains combined with Prevacent, no contigs corresponding to L1A were recovered. Similarly, in the mixture containing Fostera and the three wild-type strains, no contigs corresponding to Fostera were identified. In the mixture combining the three wild-type strains with Prime Pac, contigs corresponding to L1A and L1C.5 were not recovered. However, contigs representing the remaining components in each mixture were recovered, each with ≥98% nucleotide identity.

In the six-vaccine mixture prepared at Ct 27, Ingelvac MLV, Fostera, and Ingelvac ATP were not detected. The virus that was successfully recovered at Ct 27 was Prevacent, assembled with genome coverages 17.2%. Similar to the Ct 21 results, two assembled contigs from the non-structural region of the genome were assigned inconclusively, as they matched both Prime Pac and PRRSGard with high sequence similarity. Specifically, these contigs exhibited 99.65% and 99.83% similarity to Prime Pac and 99.50% and 99.92% similarity to PRRSGard, preventing definitive assignment (Supplementary Table).

In the two virus mixtures prepared with different Ct values, i.e., Ct 21 and Ct 27 or Ct 27 and Ct 21, for each of the viruses, 93% of the samples (28 of 30) produced contigs for both of the reference virus present in the sample, with only 6.6% of the samples (2 of 30) containing a missing virus (Table 4). A total of 472 contigs were assembled in a mixed Ct virus category with contig lengths ranging from 41 to 8,741 nucleotides. Among the assembled contigs, 448 showed nucleotide identity of at least 98% to their corresponding PRRSV-2 reference genomes. Six contigs aligned between 96 and 98% identity. Five assembly artifact contigs were detected in one mixture. The three contigs were split into two, and two contigs were split into three, which helped the unmatched (< 95% similarity) and low matching (96–98% similarity) contigs to become more than 98% matching contigs, and only 3 out of the 12 split contigs became 97% similar.

The mixture containing L1A at Ct 27 and L1H at Ct 21 generated several contaminant contigs that could not be assigned to either reference genome. One sample initially produced three contaminant contigs, none of which were detected upon repeat sequencing. A second sample initially contained five contaminant contigs; after retesting, a single contaminant contig was present. Likewise, a third sample initially produced three contaminant contigs; following retesting, no contaminant contig was recovered. However, a single assembly artifact was identified. The mixed-Ct sample containing L1C.5 at Ct 27 and L1H at Ct 21 also produced two contigs classified as assembly artifacts. One of these contigs was determined to be an artificial recombinant contig because its two segments independently aligned to the two reference viruses with greater than 98% nucleotide identity. As observed in the same-Ct experiments, genome coverage shifted in response to both input mixing ratios and Ct differences, reflecting the combined effects of viral abundance and concentration.

In the L1A (Ct 21) plus L1C.5 (Ct 27) mixtures, genome coverage for L1A was consistently higher across all dilution ratios. When the Ct values were reversed within the same lineage pair, a mirrored coverage pattern was observed (Figure 6). In the L1A (Ct 21) plus L1H (Ct 27) mixtures, L1A maintained high genome coverage across ratios, with only a modest reduction when its proportion increased from 50% to 70%. In contrast, L1H showed a marked decline in coverage as its proportion decreased from 50% to 30% and was not recovered when it comprised only 10% of the mixture. When Ct values were reversed for this lineage pair, L1H exhibited consistently higher coverage regardless of its proportion in the mixture (Figure 6). A similar pattern was observed for the L1C.5 and L1H combinations. When L1C.5 was at Ct 21, genome coverage was substantially higher and remained stable across all mixing ratios. In contrast, L1H at Ct 27 showed a steady decline in coverage despite increases in its relative proportion and was not recovered when present at 30% of the mixture. When Ct values were reversed, L1H coverage was consistently higher, with a gradual reduction as its proportion decreased (Figure 6).

**Figure 6:**
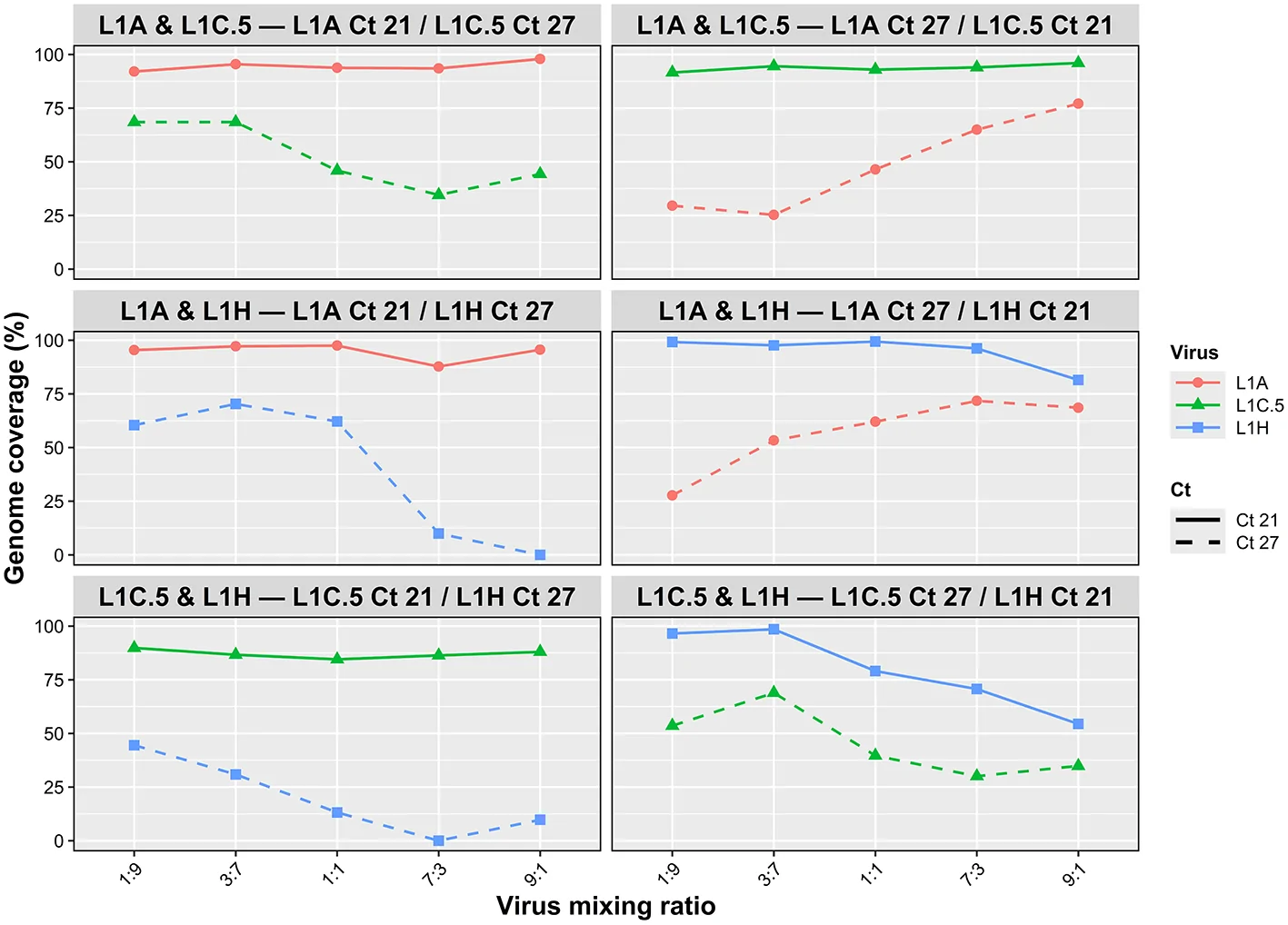
Percentage of genome coverage that was recovered from PRRSV lineages in two-virus mixtures with alternating Ct values. Each panel shows genome coverage across mixing ratios when one virus was present at Ct 21, and the paired virus at Ct 27, with Ct assignments reversed in the corresponding paired panel. Solid lines display viruses present at Ct 21, whereas dashed lines indicate viruses present at Ct 27. Virus mixture ratios are reported as first-listed virus:second-listed virus (e.g., 1:9).

## 4 Discussion

Porcine reproductive and respiratory syndrome virus remains one of the most significant health and economic challenges facing the swine industry worldwide (8). A wide range of control strategies has been implemented, including enhanced biosecurity, herd depopulation and closure, modified live virus vaccination, and the use of live virus inoculum (31–34). The selection of an appropriate control strategy critically depends on the accurate identification of the PRRSV variant (s) responsible for an outbreak. Variant identification is commonly performed using Sanger sequencing of ORF5 or next-generation sequencing (18, 35). While Sanger sequencing generally detects and reports a consensus sequence and has limited ability to identify mixed PRRSV-2 strains when present in the same sample (36). In contrast, NGS has demonstrated the ability to differentiate PRRSV-1 and PRRSV-2 when both species are present in a sample (18). Studies have shown mixed PRRSV-2 infections in 11.47% of PRRS-affected sow farm samples, and 90% of monitored farms contained at least two wild-type strains, with 40% also harboring vaccine-like strains (9, 37).

Therefore, this study was conducted to evaluate the assembled partial contig results generated by NGS and determine the effectiveness of correctly detecting a mixed infection and the ability to accurately identify the strains involved when the underlying reference genome is known, and to develop a guideline for result interpretation. To this end, nine PRRSV-2 strains were spiked into processing fluid to generate PRRSV-positive samples at Ct 21 and Ct 27. The spiked samples were mixed with different numbers of viruses, ratios, and Ct combinations to replicate field conditions in which multiple strains of wild-type and vaccine-like strains co-circulate.

In this study, when multiple PRRSV-2 strains were present in a sample with different concentrations and ratios, although whole-PRRSV genomes were not obtained for any viruses mixed in the sample, the obtained partial contigs were able to correctly identify the mix of viruses present in 89.7% of the tested samples (70 of 78). In 10.3% of samples (8 of 78) with known viruses present in the mixture, no corresponding contigs were recovered yielding missing virus (*n* = 6) or missing and inconclusive results (*n* = 2) (Table 4). Only two of the eight mixtures contain two viruses, with the remaining containing virus mixtures of four and six. This indicates NGS can reliably characterize simpler (2-virus and 3-virus) mixtures, but sensitivity decreases when four or more strains are present. Even though NGS is highly dependent on Ct value for the detection of PRRSV (18) it is also dependent on the complexity of the mixtures. Consequently, while NGS is a powerful tool for resolving most mixed PRRSV infections, interpretation should be considered with caution when complex mixtures are suspected, since some strains may go undetected. Still, in this work, most NGS reported contigs representing short genome fragments, comprising 1 to 2% of the whole-PRRSV, could be identified as similar to a reference genome present in the dilutions. When using NGS-reported results for epidemiological comparisons, caution is warranted, as comparisons can be influenced by the region of the genome examined. Regions that are more conserved across viruses may fail to differentiate, as happened with contigs from the non-structural region of Prime Pac and PRRSGard, which couldn't be distinguished from one another.

When both viruses were at Ct 21, genome coverage generally followed expected trends based on mixing proportions generally having a high percentage of genome coverage recovery for each virus. At Ct 27, genome coverage became more variable and less proportional to mixing ratios. When viruses with different Ct values were mixed, the virus at Ct 21 achieved high and stable coverage across all five ratios, while the Ct 27 virus showed reduced and more variable recovery even when it accounted for 70–90% of the mix. When a high Ct virus was present at low proportions (30–10%), two cases showed no detection of such virus, indicating that viruses with low abundance and high Ct values may go undetected by NGS. Generally, in these mixes, the proportion of the virus present in the sample does not linearly translate to coverage when Ct values are substantially different. Therefore, Ct value is a primary driver of genome recovery, outweighing proportional abundance in mixed infections.

In both the Ct 27 samples and the mixed Ct 21 and Ct 27 samples, recovery of artificial recombinant contigs was observed. These contigs initially appeared to be assembly artifacts, as they showed low nucleotide identity ( ≤ 85%) to any of the reference genomes. However, further investigation revealed that they were chimeric sequences composed of genomic regions derived from two viruses present in the mixture. Such an artificial recombinant contig may arise during library preparation as a result of *in vitro* recombination events (38) or during the assembly process (39). In the present study, it is very unlikely that these chimeras originated during the wet lab stage since no culturing of the virus took place between dilution and NGS testing. The artificial recombinant contig was very likely produced during sequencing or at the sequence assembly stage. Splitting the contigs at the recombination breakpoint and comparing them with reference strains present in the sample, and reanalyzing the resulting fragments independently, allowed such identification. Farm-specific reference viruses are of great value to epidemiological investigations. When interpreting NGS results from field samples, caution should be taken for recombination events, and further virus isolation and cell culture purification should be conducted to rule out NGS artifacts. Additionally, read-level support across the junction or targeted RT-PCR across the breakpoint would be of additional value for a direct assessment in case of recombinant virus. However in this study, read-level support across the junction was consistent with an artificial recombinant contig but was of limited value in determining whether the chimera arose during library preparation, sequencing, or assembly.

One important observation from this work is that most of the time NGS detected strains even when they accounted for only 10% of the viral population at relatively high Ct values. This confirms that mixed infections can be detected reliably at low abundance even though no whole genomes were recovered, underscoring an important point for diagnostic interpretation. Complete genomes are not strictly required to determine within-sample strain composition as long as reference genomes are known. Contigs originating from different parts of the genome carried enough discriminatory signal between the different PRRSV strains to enable accurate identification. However, in 10.3% (8 of 78) of samples, viruses known to be present in the samples were not detected, highlighting the importance of considering the potential presence of additional PRRSV strains in a sample even if not detected by NGS.

Genome coverage recovery consistently decreased as viral concentration decreased, i.e., with increasing Ct values. This aligns with established relationships whereby low Ct values yield higher sequencing depth and coverage than high Ct values (9, 18). Based on these results, an identity threshold of 98% was considered appropriate for contig classification when the viral load is high, i.e., low Ct values, while when working with lower concentration, i.e., Ct 27 and higher, contigs with a nucleotide identity of ≥96% can be indicative of the presence of the same reference virus. Nucleotide identities below 98% for samples with a Ct 21 or 96% for a Ct 27 indicate either the presence of a different virus in the sample, a potential assembly artifact, an artificial recombinant contig, or contamination. These cutoffs provide practical guidance for interpreting NGS data from PRRSV-positive field samples where Ct values span a wide range. A suggestive flowchart outlining an approach to interpreting NGS results that include contigs is provided in Figure 7.

**Figure 7:**
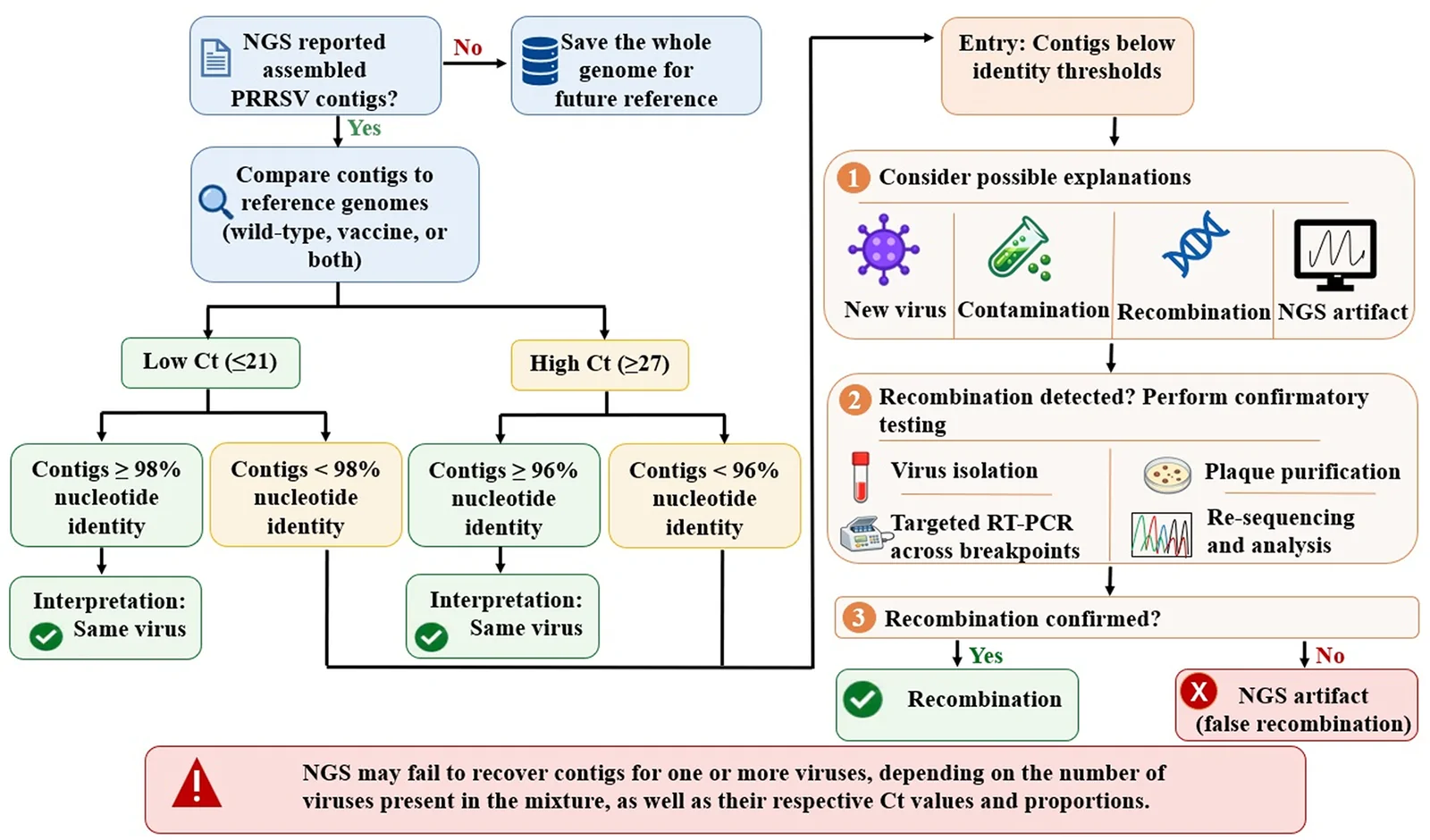
Potential approach for PRRSV NGS results interpretation.

The limitations of this study include the absence of experimental replicates for the dilutions, primarily due to the high cost associated with NGS. Even though this is a limitation, it mimics real-world situations, where a sample is taken and only one NGS run is performed to detect genetic material present in the sample. Another limitation lies in the fact that there are slight deviations of the final measured Ct values from the intended target Ct values of 21 and 27, which could contribute to slight differences in the NGS recovery. The use of an established service limits the ability to create a specific assembly pipeline for the whole PRRSV genome of the strains used in the dilutions. Better coverage could have been obtained if a specific pipeline had been developed. Extrapolation of the results encountered here to other sample types, other viral or bacterial agents, should be done with caution, since processing fluid samples are recovered from many pigs and may contain inhibitors not present in other sample types, such as serum.

The potential for NGS assembly procedures to generate artifacts is well recognized (40, 41). The process of a *de novo* assembler relies on the alignment of sequences between obtained reads to create a consensus sequence. When sequences present in the same sample are very apart like PRRSV-1 and PRRSV-2, it is well recognized that it can yield complete genomes for both virus though NGS technique (18, 42). Errors can occur due to reads being incorrectly discarded as mistakes or repeats, or joined up in the wrong places or orientations (43). In this study, most of the sequences (except Prime Pac and PRRSGard) were far apart, and assembly error was very likely low. However, mismatches were enough to create eight artifacts that led to artificial recombinant contigs. When the sequences are more similar, the creation of assembly artifacts could be more frequent and more difficult to detect without further analysis, sample purification, or specialized software like RDP. Another limitation of this study is that the use of more closely related isolates would have allowed for better calibration in these situations. This limitation underscores the need for cautious interpretation of PRRSV NGS results in epidemiological investigations.

## Data Availability

The raw sequencing data presented in the study are deposited in the NCBI Sequence Read Archive repository, BioProject accession number PRJNA1502979 (https://www.ncbi.nlm.nih.gov/sra/PRJNA1502979).
